# Cost-effectiveness analysis of implementing a field hospital in a soccer stadium during a pandemic

**DOI:** 10.3389/fpubh.2025.1513709

**Published:** 2025-04-22

**Authors:** Sabrina Bernardez-Pereira, Guilherme de Paula Pinto Schettino, Alexandre R. Marra, Kaue Capellato Junqueira Parreira, Fabio de Castro Jorge Racy, Rose Mara Miranda, Artur Martins Codeço, Michele Jaures, João Gabriel Dias Pagliuso, Miguel Cendoroglo Neto, Claudia Regina Laselva, Sidney Klajner, Vanessa Damazio Teich, Danilo Nascimento Giliberti, Takaaki Kobayashi, Michael B. Edmond, Daniel Tavares Malheiro

**Affiliations:** ^1^Hospital Israelita Albert Einstein, São Paulo, SP, Brazil; ^2^Department of Internal Medicine, University of Iowa Carver College of Medicine, Iowa, IA, United States; ^3^West Virginia University School of Medicine, Morgantown, WV, United States

**Keywords:** field hospital, COVID-19, cost-effectiveness analysis, health-adjusted life years, pandemic

## Abstract

**Introduction:**

Field hospitals, following the Fangcang Shelter Hospital model, were critical during the global COVID-19 pandemic to alleviate the strain on overwhelmed healthcare systems. Despite their widespread adoption, concerns persist regarding their efficacy and cost-effectiveness. This study aimed to assess the impact of the Pacaembu Field Hospital in São Paulo, Brazil during the COVID-19 pandemic, specifically focusing on lives saved and the associated public health costs.

**Methods:**

This retrospective cohort study was conducted April 6 to June 29. The 200-bed field hospital, a collaboration between Sociedade Beneficente Israelita Brasileira Albert Einstein and the São Paulo City Hall, São Paulo Municipal Health Departament, operated at Pacaembu Stadium and admitted. Adult patients with mild to moderate COVID-19. Electronic health records provided comprehensive data on demographics, clinical outcomes, and resource utilization. The mortality rate among field hospital patients was compared to that of two groups: I-confirmed COVID-19 cases in São Paulo, and II-severe acute respiratory syndrome patients with COVID-19 in São Paulo.

**Results:**

A total of 152,928 COVID-19 cases were confirmed in São Paulo, with 20,603 patients hospitalized for ARDS and 1,499 patients admitted to the Pacaembu Field Hospital for mild to moderate disease. The median age of Pacaembu patients was 57 years (IQR 46–67), with 43.8% aged 60 or older. Lung disease was the most common comorbidity, affecting 31.0% of cases. The median length of stay was 4.2 days, and 14.2% of patients required intensive care, with 7.9% needing mechanical ventilation. The hospital had a survival rate of 99.8%. The cost per year of life saved, adjusted for gender, was R$44,243.02 (US$8,208.35). In the most favorable scenario, approximately 200 lives were saved, with a cost of R$5,640.92 (US$1,046.55) per life saved for both genders. In the least favorable scenario, around 50 lives were saved, with a cost of R$36,863.48 (US$6,839.24) per life saved for both genders, all within cost-effectiveness thresholds.

**Conclusion:**

The Pacaembu Field Hospital played a crucial role in saving lives during the initial COVID-19 wave, highlighting the importance of ongoing evaluation and resource optimization in field hospital strategies for an effective pandemic response.

## Introduction

The use of field hospitals to augment the healthcare system as a solution to deal with an increase in patients due to wars, outbreaks of pandemic diseases or natural disasters has long been practiced. For example, during World War I, they provided immediate care to wounded soldiers, significantly improving survival rates. Similarly, during the 2003 SARS outbreak, field hospitals in Hong Kong and Toronto helped isolate infected individuals, preventing widespread transmission. In the aftermath of the 2004 Indian Ocean tsunami, they provided vital care to survivors and prevented further outbreaks (1–3).

Amid the devastating effects caused by the COVID-19 pandemic around the world, including the scarcity of hospital beds and resources due to high viral transmissibility, the construction of field hospitals was a solution found to improve efficiency in combating the disease (4, 5).

Professor Wang Chen in Wuhan, China, first proposed a new concept of field hospital - the Fangcang Shelter Hospital - in February 2020. Fangcang, which means “square cabin” in Chinese, refers to a new concept of temporary hospitals, which are constructed by converting public places such as stadiums and exhibition centers into healthcare facilities for isolating patients with mild to moderate symptoms of an infectious disease, while providing medical care and disease monitoring (6).

Despite the implementation of these types of hospitals around the world, there is controversy about their true cost-effectiveness in practice and little has been investigated about how much this strategy has saved lives (7). The debate largely stems from the significant costs involved in establishing and maintaining these temporary facilities, which include infrastructure, medical personnel, and resources. Some argue that the cost might outweigh the benefits, especially when compared to alternative public health interventions like strengthening existing healthcare facilities. However, others contend that field hospitals provide an essential lifeline in emergencies, offering a rapid response to surges in patient volume, and could potentially save lives by preventing healthcare systems from becoming overwhelmed (8).

This research aimed to assess the impact of field hospitals in addressing the COVID-19 crisis in Brazil, particularly focusing on the implementation of the Pacaembu Field Hospital in São Paulo. The study evaluated the years of life saved, the number of patients saved, and the economic feasibility of establishing such facilities. With a broader understanding of their effectiveness, this research can inform future emergency healthcare responses, contributing to the optimization of public health interventions in the context of pandemics or other health emergencies. Comparing the outcomes of this field hospital with traditional healthcare systems could provide valuable insights into how these facilities can be integrated into national healthcare strategies.

## Materials and methods

This study follows the CHEERS 2022 guidelines to ensure transparency and rigor in conducting and reporting the economic analysis. The CHEERS 2022 checklist has been completed and is available as Supplementary material 1.

### Study design, setting, perspective, inclusion and exclusion criteria

This retrospective cohort study was conducted at the Pacaembu Field Hospital in São Paulo, Brazil. The target population included all patients admitted during the first wave of COVID-19, from April 6 to June 29, 2020, with suspected or confirmed cases of mild to moderate disease.

The study adopts the perspective of the Brazilian public healthcare system, evaluating costs incurred by both São Paulo City Hall and Hospital Israelita Albert Einstein in operating the field hospital. The economic evaluation aligns with guidelines from the National Commission for the Incorporation of Technologies in the Unified Health System (CONITEC), which establishes cost-effectiveness thresholds for health interventions within the Unified Health System (SUS).

Additionally, patients transferred from the field hospital to other hospitals managed by the Sociedade Beneficente Israelita Brasileira Albert Einstein (SBIBAE) due to clinical deterioration were monitored for discharge outcomes, enabling an assessment of the mortality rate among those initially treated at the Pacaembu Field Hospital.

### Pacaembu field hospital

In mid-March 2020, due to the high occupancy of hospital beds in São Paulo’s public healthcare system with the number of individuals with Acute Respiratory Distress Syndrome (ARDS) doubling every 2.17 days, there was a real and imminent threat of saturation and collapse of the municipal hospital system. This could have happened if measures were not taken to reduce the spread of the new coronavirus and increase the capacity of hospital beds, particularly in intensive care units, to care for the most critical patients.

To address this challenge, the Pacaembu Field Hospital was established on April 6, 2020 in São Paulo through a partnership between the SBIBAE and São Paulo City Hall São Paulo, Municipal Health Departament. This facility was the first of its kind in the country, aiming to alleviate the burden on the health system and serving as a model for similar initiatives in other states. The 6,300 m^2^ hospital was constructed on an emergency basis in just 10 days and was specifically designed to accommodate patients with suspected or confirmed COVID-19 presenting with mild to moderate severity. These patients were referred after evaluation in basic health units of the Unified Health System (SUS) of Brazil, representing those who should not stay at home, but who were not severe enough to occupy traditional hospital beds, which were reserved for the most critical cases.

**Figure 1:**
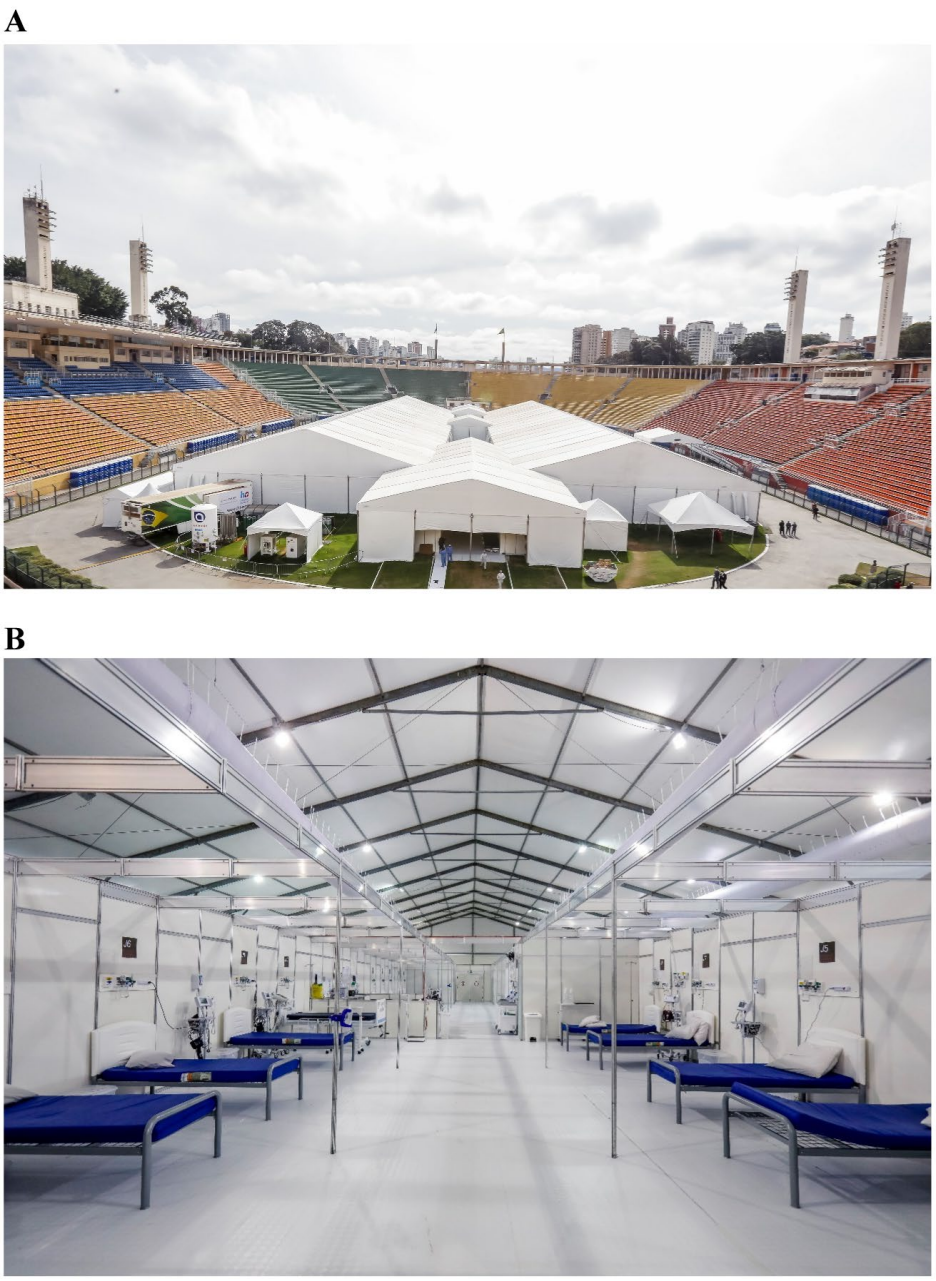
**(A)** External view of Pacaembu field hospital. **(B)** Internal view of Pacaembu field hospital.

The hospital was set up on the entire lawn of the Pacaembu Soccer Stadium and comprised two large tents. Inside, there were 200 beds, including 16 equipped with intensive care capabilities, distributed across 10 wards, each containing 20 beds and a dedicated nursing station. Patients with a clinical suspicion of COVID-19, while awaiting laboratory confirmation, were admitted to a designated ward. Laboratory confirmation was performed using RT-PCR with samples obtained from the upper respiratory tract, specifically nasopharyngeal and oropharyngeal swabs. These swabs were stored in sterile tubes containing saline solution. Sample detection was conducted using either an in-house technique or the XGen Master COVID-19 Mobius method. The wards were segregated by gender and equipped with bathrooms that included sinks, toilets, and showers (Figures 1A,B).

The operation of the hospital required the dedication of 588 professionals, comprising a multidisciplinary team that included doctors, nurses, nursing assistants, pharmacists, biomedical scientists, physiotherapists, social workers, and psychologists, as well as personnel from various support areas such as administration, housekeeping, logistics, and security. Clinical protocols were established for both diagnosis and treatment, with all healthcare professionals receiving appropriate training to ensure standardized care. To support diagnostic procedures, the hospital had mobile X-ray machines, ultrasound devices, and a CT scanner installed in a trailer.

City Hall was responsible for assembling the hospital structure, while Hospital Israelita Albert Einstein managed the hospital’s operation, including human resources and medical equipment. With a decline in COVID-19 cases, the hospital ceased operations on June 30, 2020, operating only during the first wave of the pandemic.

### Study time horizon

The time horizon for this study spans from the admission of patients to the Pacaembu Field Hospital, between April 6 and June 29, 2020, until hospital discharge or final clinical outcome, including potential transfers to other healthcare facilities. This period was chosen to fully capture the care cycle provided by the field hospital, allowing for a comprehensive assessment of the costs and clinical outcomes associated with the services delivered.

### Admission criteria to Pacaembu field hospital

Admission requests for the Pacaembu Field Hospital were screened by the Municipal Regulation Service and approved by the hospital’s internal regulation team based on predefined criteria. Patient transport to the facility was exclusively arranged through ambulance services, with no provision for unscheduled walk-ins.

All patients admitted were transferred by ambulance from public outpatients’ unit or hospitals. Upon arrival, they were directed either to a general ward or to a stabilization room, based on specific clinical criteria (Tables 1, 2).

**Table 1:** Ward admission criteria.

Criterion	Description
Age	≥18 years old
Length of Stay	≥24 h
Respiratory Rate	≤24 breaths per minute
Oxygen Saturation	≥88% on room air or ≥ 94% with supplemental O2 < 4 L/min
Hemodynamic Stability	No use of vasoactive drugs (SBP >90 mmHg or MAP >65 mmHg) for >24 h
Glasgow Coma Scale	≥13
Weight	<110 Kg
Onset of Symptoms	>24 h
Intubation	Not intubated or requiring non-invasive ventilation for >48 h

MAP, Mean arterial pressure; SBP, Systolic blood pressure. Exclusion criteria included: (1) active oncologic or onco-hematologic disease; (2) chemotherapy within 30 days and/or neutropenia (WBC < 1,000 cells/μL); (3) Transplant recipient or use of immunosuppressants; (4) Pregnancy; (5) Renal Replacement Therapy; (6) Untreated tuberculosis; (7) Hyperactive delirium; (8) Psychiatric or behavioral disorders; (9) Contact isolation due to multidrug-resistant bacteria; (10) Confirmed diagnosis of influenza; (11) Liver failure and/or cirrhosis; (12) individuals living with HIV.

**Table 2:** Stabilization room admission criteria.

Criterion	Description
Oxygen Saturation	<94% with non-rebreather mask O2 delivery above 10 L/min or oxygen saturation < 94% with O2 up to 5 L/min (nasal cannula or non-rebreather mask), with signs of respiratory distress
Ventilation Needs	Need for invasive or noninvasive ventilatory support
Hemodynamic Instability	SBP <90 mmHg or MAP <65 mmHg, or lactate >36 mg/dL
Glasgow Coma Scale	<13 or acute organ dysfunction

MAP, Mean arterial pressure; SBP, Systolic blood pressure.

### Discharge criteria

The discharge criteria for the Pacaembu Field Hospital included improvement of symptoms; absence of fever >48 h; reduction of inflammatory markers; oxygen saturation ≥ 93% and respiratory rate < 24 breaths per minute (room air).

### Hospital transfer from Pacaembu field hospital to a general hospital

The criteria for transfer to highly complex health care facilities included: (1) need for progressive oxygen supplementation >10 L/min; (2) respiratory rate > 30 breaths per minute associated with signs of respiratory distress; (3) need for noninvasive ventilation and mechanical ventilation; (4) persistent hemodynamic instability; (5) need for vasopressor drugs; (6) Glasgow coma scale <13; (7) acid–base imbalance characterized by pH <7.35 and PaCO2 > 50 mmHg.

The hospitals to which these patients were transferred were either public or private institutions, as registered in the Health Service Offers Regulation Center (CROSS) network. This system includes various hospitals that can accept patients in need of transfer. Of the 39 hospitals in the CROSS network where patients were transferred, patient records were accessible for three facilities managed by SBIBAE: Hospital Israelita Albert Einstein Morumbi (HIAE), Hospital Municipal Dr. Moysés Deutsch (HMDMD), and Hospital Municipal Vila Santa Catarina (HMVSC). Among these, HMDMD and HMVSC were part of the public health network, while HIAE belonged to the private sector.

### São Paulo state data analysis system foundation (SEADE)

Data from SEADE were utilized to analyze the baseline characteristics, clinical features, and mortality of confirmed COVID-19 cases in the state of São Paulo, as well as those who developed ARDS compared with Pacaembu Field Hospital Results.

SEADE, a foundation linked to the Brazilian Secretariat of Finance and Planning, is a national reference center for the production and dissemination of socioeconomic and demographic analyses and statistics. SEADE has become a reliable and always up-to-date source of data about the state of São Paulo. The SEADE Coronavirus dataset includes data on cases, deaths, and hospitalizations related to COVID-19 in the state of São Paulo. Daily data since the beginning of the COVID-19 pandemic are available in the repository and have been updated up to November 18, 2023 (9).

### Selection of outcomes

The selected outcomes include the mortality rate and the average length of hospital stay among patients treated at the Pacaembu Field Hospital, as well as the estimated number of lives and life-years saved.

### Collection data

Data from Pacaembu Field Hospital was collected through queries to the information system (Business Intelligence - BI) and electronic health records. The collected data include demographic information, clinical outcome data (length of stay, mortality rate), and cost data of resources used.

### Statistical analysis and economic evaluation

The mortality rates among Pacaembu Field Hospital patients were evaluated in comparison to two scenarios: I confirmed COVID-19 cases in São Paulo, and II severe acute respiratory syndrome patients with COVID-19 in São Paulo.

The age-and sex-adjusted mortality results were compared to the data for the total population of confirmed COVID-19 cases and ARDS cases in the state of São Paulo, as provided by the São Paulo SEADE. Additionally, the lives saved were calculated considering the number of patients in the city of São Paulo (the total confirmed COVID-19 population and ARDS cases, individually) and the projected deaths estimated from the transferred patients, resulting in the projected deaths if both of the populations had the mortality rate of Pacaembu. Then the overall number was multiplied by the life expectancy for that sex and age. Schematically:

**Lives saved** = [Projected mortality rate from Pacaembu * confirmed COVID-19 or ARDS patients - Projected deaths from Pacaembu], and

**Years of life saved** = [Lives saved * life expectancy for sex and age].

The overall mortality rate for COVID-19 in the city of São Paulo as well as those patients who developed ARDS was stratified by age group. As reliable databases by health facility in São Paulo city are not available, the mortality rate among those patients transferred from the Pacaembu Field Hospital to other hospitals was performed by extrapolation with the mortality rate of patients transferred to the general hospitals managed by SBIBAE.

From this data, lives saved at the Pacaembu Field Hospital were projected by age and sex. Using the life expectancy of each group, based on Brazilian Institute of Geography and Statistics 2020 data (10), the years of life saved were determined. Additionally, further analysis was conducted, including age group for model adjustment. The variable representing the age was discretized and grouped into 14 groups starting from 15 years old and with 5 years each; people aged 80 or more years were grouped into one group. For these groups, life expectancy was considered as the median value of each interval. For the calculation of life of years saved, half-way values were rounded up.

To compare the demographic characteristics of the patients admitted in different hospitals, the Pearson’s Chi-square and Fisher’s exact test were used for categorical variables and were summarized as counts and percentages. Normality assumptions were tested by the Anderson-Darling normality test, such that if evidence against the data following a normal distribution was significative, the Wilcoxon-Mann–Whitney U test was used for continuous variables, otherwise, Student’s t-test was used (11). Both were expressed as medians with IQR (Interquartile Range). Also, values were converted from Brazil’s currency ‘Real’ (R$) to US dollars (US$). A mean value of the US dollar was obtained from the Central Bank of Brazil based on the daily exchange rates during the entire period of Pacaembu’s utilization, which spanned from April 6, 2020, to June 29, 2020 (US$1 = R$5.39).

At the time the field hospital was implemented, local hospitals lacked the capacity to handle the demand for COVID-19 cases. As a result, the comparison was made against a ‘no treatment’ scenario. The cost per incremental life saved was calculated by dividing the difference in costs between treating patients at the Pacaembu Field Hospital and providing no treatment, by the difference in lives saved between the two approaches. Similarly, the cost per incremental year of life saved was determined by multiplying the lives saved by the average life expectancy and dividing the incremental cost by this value. To ensure a more real comparison, effectiveness was evaluated by comparing the mortality rates and estimated years of life saved at the field hospital with the two previously established scenarios.

In Brazil, the definition of cost-effectiveness for a health technology generally follows criteria established by institutions such as the National Commission for the Incorporation of Technologies in the Unified Health System (CONITEC). A technology is considered cost-effective if the additional cost per unit of benefit falls within an acceptable range for the health system. According to a document published by the Brazilian Ministry of Health in 2022, the threshold for cost-effectiveness is set at a maximum of 0.87 Gross Domestic Product (GDP) per capita per year of life gained, which corresponds to R$ 50.193,72 in 2023 (12).

All analysis were performed using the Knime Analytics Platform (version 4.4.1) and R Statistical Language (version 4.2.0). R packages: tidyverse (version 1.3.2) for data transformation and manipulation; gtsummary (version 1.7.1) for tables and statistical tests; nortest (version 1.0.4) for data normality evaluation were used.

### Ethics

This study was approved by the Ethics Committee of the Hospital Israelita Albert Einstein (ECP) under the protocol number 6.028.323, CAAE: 66954722.9.0000.0071. Clinical trial number: not applicable. All the procedures in this study were in accordance with the 1975 Helsinki Declaration, updated in 2013. The study was exempted from the consent form requirement by the Ethics committee for the following reasons: (i) as it is a retrospective observational study, which will only use information from medical records, institutional information systems and/or other sources of data and clinical information available at the institution with no provision for the use of biological material; (ii) because all data will be handled and analyzed anonymously, without nominal identification of research participants; (iii) because the results resulting from the study will be presented in aggregate form, not allowing the individual identification of participants; and (iv) because it is a non-interventional study (without clinical interventions) and without changes/influences in the routine/treatment of the research participant and, consequently, without adding risks or harm to the well-being of the members. Furthermore, we will not be able to obtain consent from all participants in this research.

## Results

### Baseline and clinical characteristics

A total of 152,928 patients were confirmed with COVID-19 in the city of São Paulo, with 20,603 patients being hospitalized with ARDS and 1,499 patients being admitted to the Pacaembu Field Hospital with a diagnosis of mild to moderate COVID-19 (Table 3).

**Table 3:** Baseline characteristics of study population.

Variables	ARDS patients *N* = 20,603^*^	Pacaembu patients *N* = 1,499	*p*-value
Month, n (%)			**<0.001** ^**†**^
April, 2020	8,072 (39.2%)	373 (24.9%)	
May, 2020	10,602 (51.5%)	801 (53.4%)	
June, 2020	1,929 (9.4%)	325 (21.7%)	
Sex, n (%)			0.17*^†^*
Male	11,471 (55.7%)	807 (53.8%)	
Age, Median(P25-P75)	58 (45–72)	57 (46–67)	**<0.001** ^**‡**^
Age Groups, n (%)			**<0.001** ^**§**^
15–19 years	45 (0.2%)	1 (0.1%)	
20–24 years	262 (1.3%)	13 (0.9%)	
25–29 years	551 (2.7%)	39 (2.6%)	
30–34 years	963 (4.7%)	62 (4.1%)	
35–39 years	1,448 (7.0%)	96 (6.4%)	
40–44 years	1,660 (8.1%)	128 (8.5%)	
45–49 years	1,780 (8.6%)	154 (10.3%)	
50–54 years	1,999 (9.7%)	170 (11.3%)	
55–59 years	2,049 (9.9%)	179 (11.9%)	
60–64 years	1,908 (9.3%)	205 (13.7%)	
65–69 years	1,827 (8.9%)	157 (10.5%)	
70–74 years	1,740 (8.4%)	145 (9.7%)	
75–79 years	1,443 (7.0%)	92 (6.1%)	
80+ years	2,928 (14.2%)	58 (3.9%)	
Lung Diseases, n (%)			**<0.001** ^**†**^
No	5,844 (28.4%)	1,034 (69.0%)	
Yes	874 (4.2%)	465 (31.0%)	
Missing	13,885 (67.4%)	0 (0%)	
Diabetes, n (%)			**<0.001** ^**†**^
No	3,843 (18.7%)	1,334 (89.0%)	
Yes	5,354 (26.0%)	165 (11.0%)	
Missing	11,406 (55.4%)	0 (0%)	
Cardiopathy, n (%)			**<0.001** ^**†**^
No	2,696 (13.1%)	1,212 (80.9%)	
Yes	7,986 (38.8%)	287 (19.1%)	
Missing	9,921 (48.2%)	0 (0%)	
Obesity, n (%)			**<0.001** ^**†**^
No	5,366 (26.0%)	1,400 (93.4%)	
Yes	1,217 (5.9%)	99 (6.6%)	
Missing	14,020 (68.0%)	0 (0%)	
Chronic Kidney Diseases, n (%)			**<0.001** ^**†**^
No	5,637 (27.4%)	1,429 (95.3%)	
Yes	1,090 (5.3%)	70 (4.7%)	
Missing	13,876 (67.3%)	0 (0%)	
Asthma, n (%)			**<0.001** ^**†**^
No	5,916 (28.7%)	1,473 (98.3%)	
Yes	661 (3.2%)	26 (1.7%)	
Missing	14,026 (68.1%)	0 (0%)	
Liver Diseases, n (%)			**<0.001** ^**†**^
No	6,145 (29.8%)	1,469 (98.0%)	
Yes	238 (1.2%)	30 (2.0%)	
Missing	14,220 (69.0%)	0 (0%)	
Neurological Disorders, n (%)			**<0.001** ^**†**^
No	5,770 (28.0%)	1,487 (99.2%)	
Yes	1,215 (5.9%)	12 (0.8%)	
Missing	13,618 (66.1%)	0 (0%)	
Hematological Diseases, n (%)			**<0.001** ^**†**^
No	6,184 (30.0%)	1,488 (99.3%)	
Yes	234 (1.1%)	11 (0.7%)	
Missing	14,185 (68.8%)	0 (0%)	
Pregnant And Postpartum Women, n (%)			**<0.001** ^**‖**^
No	6,075 (29.5%)	1,497 (99.9%)	
Yes	59 (0.3%)	2 (0.1%)	
Missing	14,469 (70.2%)	0 (0%)	
Deaths, n (%)	4,777 (23.2%)	55 (3.7%)	**<0.001** ^**†**^
Length of Stay (days), Median(P25-P75)	6.0 (2.0–11.0)	4.2 (2.6–6.9)	**<0.001** ^**‡**^
Missing	17,766 (86.2%)	0 (0%)	

*ARDS, Acute Respiratory Distress Syndrome. †Pearson’s Chi-squared test. ‡ Wilcoxon rank sum test. § Fisher’s Exact Test for Count Data with simulated *p*-value (based on 2,000 replicates). ‖ Fisher’s exact test. Bold values indicate statistically significant differences (*p* < 0.05).

Of those cases admitted to Pacaembu Field Hospital, 53.8% were males and median age was 57 years [interquartile range (IQR), 46–67], [43.8% older adult people ≥60 years]. Among these patients, lung disease prevailed with 31.0% of the cases. The median length of stay was 4.2 days. Of the total, 213 (14.2%) patients used the stabilization room (intensive care structure). Of these, 119 (7.9%) required mechanical ventilation. Concerning in-hospital outcomes, 1,197 patients were discharged from the Pacaembu Field Hospital, 299 patients were transferred to other hospitals due to the severity of their cases (116 patients were transferred to hospitals managed by the Sociedade Beneficente Israelita Brasileira Albert Einstein (SBIBAE) whereas 183 patients were transferred to other hospitals in the city of São Paulo). The survival rate was 99.8%, with 3 deaths occurring within the unit (Table 3). Of the 116 patients transferred from Pacaembu Field Hospital to hospitals managed by SBIBAE, 52 patients died. On the other hand, a total of 20,603 patients were hospitalized in the city of São Paulo with a diagnosis of ARDS. The median age was similar to the Pacaembu Field Hospital cases of 58 years IQR[45–72] and the most prevalent comorbidities were heart disease (38.8%) and diabetes (26.0%). A total of 4,777 deaths (23.2%) occurred in this population.

### Mortality rate by age group

Table 4 compares mortality rates by age group among three cohorts: confirmed COVID-19 cases, ARDS patients, and Pacaembu Field Hospital patients. Mortality rates generally increase with age across all groups, with the highest rates observed in the 80+ age group - 42.9% for confirmed COVID-19 cases, 48.8% for ARDS patients, and 10.3% for Pacaembu patients. Overall, the Pacaembu group exhibited lower mortality rates across most age groups compared to the other two groups, with an overall mortality of 3.7%, versus 5.9% in confirmed cases and 23.2% in ARDS patients.

**Table 4:** Mortality rate by age group.

Age	COVID-19 confirmed cases *N* = 152,928	ARDS patients *N* = 20,603	Pacaembu patients *N* = 1,499
	Deaths/Population (%)	Deaths/Population (%)	Deaths/Population (%)
15–19 years	3/2361 (0.1)	6/45 (13.3)	0/1 (0)
20–24 years	23/10649 (0.2)	9/262 (3.4)	0/13 (0)
25–29 years	44/14298 (0.3)	21/551 (3.8)	0/39 (0)
30–34 years	114/18056 (0.6)	73/963 (7.6)	0/62 (0)
35–39 years	178/20368 (0.9)	109/1448 (7.5)	1/96 (1)
40–44 years	249/18747 (1.3)	135/1660 (8.1)	1/128 (0.8)
45–49 years	316/15656 (2)	168/1780 (9.4)	1/154 (0.6)
50–54 years	525/13328 (3.9)	289/1999 (14.5)	4/170 (2.4)
55–59 years	698/10803 (6.5)	378/2049 (18.4)	5/179 (2.8)
60–64 years	912/8055 (11.3)	469/1908 (24.6)	8/205 (3.9)
65–69 years	1064/6151 (17.3)	553/1827 (30.3)	6/157 (3.8)
70–74 years	1225/4774 (25.7)	617/1740 (35.5)	15/145 (10.3)
75–79 years	1047/3421 (30.6)	520/1443 (36)	8/92 (8.7)
80+ years	2683/6261 (42.9)	1430/2928 (48.8)	6/58 (10.3)
Total	9081/152928 (5.9)	4777/20603 (23.2)	55/1499 (3.7)

### Economic evaluation-lives saved and life-years saved

In the scenario I, which compares confirmed cases of COVID-19 in the state of São Paulo, the number of lives and years of life saved promoted by the opening of Pacaembu Field Hospital would be 29 and 436.4 for men and 12 and 24 for women. When adjusting by sex, 44 lives and 539 years would be saved (Table 5). Grouping by age, these numbers were 33 and 498.4 for men and 14 and 57 for women; adjusting by sex, 48 lives and 646.9 years would be saved (Table 6).

**Table 5:** Scenario I—COVID-19 confirmed in health network of the state of São Paulo vs. Pacaembu field hospital by sex and age.

Cost-effectiveness metrics	Male (*N* = 807)	Female (*N* = 692)	All adjusted by sex (*N* = 1,499)
Total Lives Saved	29	12	44
Total Years of Life Saved	436.4	24	539
Cost of Pacaembu Field Hospital	R$ 12,829,678.25 (US$ 2,380,274.26)	R$ 11,017,307.34 (US$ 2,044,027.34)	R$ 23,846,985.59 (US$ 4,424,301.59)
Cost per Incremental Life Saved	R$ 442,402.70 (US$ 82,078.42)	R$ 918,105.95 (US$ 170,335.61)	R$ 541,976.95 (US$ 100,552.31)
Cost per Incremental Year of Life Saved	R$ 29,398.90 (US$ 5,454.34)	R$ 459,054.47 (US$ 85,167.81)	R$ 44,243.02 (US$ 8,208.35)

**Table 6:** Scenario I—COVID-19 confirmed in health network of the state of São Paulo vs. Pacaembu field hospital by sex and age group.

Cost-effectiveness metrics	Male (*N* = 807)	Female (*N* = 692)	All adjusted by sex (*N* = 1,499)
Total Lives Saved	33	14	48
Total Years of Life Saved	498.4	57	646.9
Cost of Pacaembu Field Hospital	R$ 12,829,678.25 (US$ 2,380,274.26)	R$ 11,017,307.34 (US$ 2,044,027.34)	R$ 23,846,985.59 (US$ 4,424,301.59)
Cost per Incremental Life Saved	R$ 388,778.13 (US$ 72,129.52)	R$ 786,950.52 (US$ 146,001.95)	R$ 496,812.20 (US$ 92,172.95)
Cost per Incremental Year of Life Saved	R$ 25,741.73 (US$ 4,775.83)	R$ 193,286.09 (US$ 35,860.13)	R$ 36,863.48 (US$ 6,839.24)

In the scenario II, in comparison to ARDS cases in São Paulo, the Pacaembu Field Hospital resulted in a total of 61 and 44 lives saved, and 1,252.5 and 872.3 years of life saved, for men and women, respectively, when considering life expectancy stratified by sex. When adjusting by sex, due to the different mortality rates and using Brazilian Institute of Geography and Statisticss’ complete life table for both sexes, 158 lives were saved, representing 3,332.1 years of life (Table 7). When grouping by age, 102 and 83 lives were saved representing 2,192.8 and 1,783.9 years of life saved for men and women, respectively; adjusting by sex, these numbers are 190 and 4,227.5 (Table 8).

**Table 7:** Scenario II—Acute respiratory distress syndrome vs. Pacaembu field hospital by sex and age.

Cost-effectiveness metrics	Male (*N* = 807)	Female (*N* = 692)	All adjusted by sex (*N* = 1,499)
Total Lives Saved	61	44	158
Total Years of Life Saved	1,252.5	872.3	3,332.1
Cost of Pacaembu Field Hospital	R$ 12,829,678.25 (US$ 2,380,274.26)	R$ 11,017,307.34 (US$ 2,044,027.34)	R$ 23,846,985.59 (US$ 4,424,301.59)
Cost per Incremental Life Saved	R$ 210,322.59 (US$ 39,020.89)	R$ 250,393.35 (US$ 46,455.17)	R$ 150,930.29 (US$ 28,001.91)
Cost per Incremental Year of Life Saved	R$ 10,243.26 (US$ 1,900.42)	R$ 12,630.18 (US$ 2,343.26)	R$ 7,156.74 (US$ 1,327.78)

**Table 8:** Scenario II—Acute respiratory distress syndrome vs. Pacaembu field hospital by sex and age group.

Cost-effectiveness metrics	Male (*N* = 807)	Female (*N* = 692)	All adjusted by sex (*N* = 1,499)
Total Lives Saved	102	83	190
Total Years of Life Saved	2,192.8	1,783.9	4,227.5
Cost of Pacaembu Field Hospital	R$ 12,829,678.25 (US$ 2,380,274.26)	R$ 11,017,307.34 (US$ 2,044,027.34)	R$ 23,846,985.59 (US$ 4,424,301.59)
Cost per Incremental Life Saved	R$ 125,781.16 (US$ 23,336.02)	R$ 132,738.64 (US$ 24,626.84)	R$ 125,510.45 (US$ 23,285.80)
Cost per Incremental Year of Life Saved	R$ 5,850.82 (US$ 1,085.50)	R$ 6,175.97 (US$ 1,145.82)	R$ 5,640.92 (US$ 1,046.55)

### Cost of the project execution

The initial projected cost of the Pacaembu Field Hospital was R$28.6 million (US$5.30 million), including both initial investment and operating costs. Of this amount, the final cost was R$23,846,985.59 million (US$4,424,301.59) to the public coffers, in addition to a cost of R$7 million (US$ 1.29 million) in equipment being provided by Hospital Israelita Albert Einstein. Upon the closing of the temporary hospital’s operations, all the equipment used was donated to three municipal hospitals, which were concentrated in the area with the highest mortality rate from COVID-19 in the state of São Paulo.

### Cost-effectiveness analysis

Scenario I estimate a cost per incremental life saved of R$442,402.70 (US$82,078.42) and R$918,108.95 (US$170,335.61) with a cost per incremental year of life saved of R$29,398.90 (US$5,454.34) and R$459,054.47 (US$85,167.81), respectively for males and females. Adjusting by sex, cost per incremental life saved was R$541,976.95 (US$100,552.31) and cost per incremental year of life saved of R$44,243.02 (US$8,208.35; Table 5). Adjusting by age group, the cost per incremental life and the cost per life year saved was, respectively, R$496,812.20 (US$92,172.95) and R$36,863.48 (US$6,839.24) for both sexes (Table 6).

The same analysis in scenario II estimates the cost per incremental life saved was R$210,322.90 (US$39,020.89) and R$250,393.35 (US$46,455.17) with a cost per incremental year of life saved of R$10,243.26 (US$1,900.42) and R$12,630.18 (US$2,343.26) for men and women, respectively. Adjusting by sex, these numbers are R$150,930.29 (US$28,001.91) and R$7,156.74 (US$1,327.78; Table 7). Adjusting by age group, the cost per incremental life saved and the cost per incremental year of life saved was, respectively, R$125,510.45 (US$23,285.80) and R$5,640.92 (US$1,046.55) for both sexes (Table 8).

## Discussion

This study presents the first cost-effectiveness analysis, based on both lives saved and years of life saved, of a temporary hospital during the COVID-19 pandemic. The establishment of a field hospital in São Paulo resulted in more than 150 lives saved over the first 3 months of the pandemic in the best-case scenario, and nearly 50 lives saved in the worst-case scenario. The cost per year of life saved, adjusted for sex, was R$44,243.02 (US$8,208.35). When analyzed by age group, nearly 200 lives were saved in the best-case scenario, with a cost per life of R$5,640.92 (US$1,046.55) for both sexes combined. In the worst-case scenario, nearly 50 lives were saved, at a cost of R$36,863.48 (US$6,839.24) per life saved for both sexes.

The concept of the Fang Shelter Hospital originated in Wuhan, China, during the COVID-19 pandemic as a response to the public health emergency characterized by a rapid surge in cases and a shortage of hospital beds. The primary objective of this type of facility is to provide a rapidly constructed healthcare setting, within a matter of days, for the early treatment of patients with low acuity, who can be transferred to more advanced hospitals should their clinical condition deteriorate (5, 6). Following the outbreak of the pandemic, several studies published in the literature reported on the experiences of various countries in converting public spaces, such as exhibition halls or basketball courts, into temporary field hospitals (13–16).

According to the National Register of Health Facilities (CNES), in September 2020, there were 214 Field Hospitals registered in Brazil, distributed across 23 states of the federation. These field hospitals operated within the public health system with a “regulated entry” model, meaning they were designated to receive patients with respiratory symptoms who were referred by “open door” health services (triage points within the health system). They were characterized by providing low to medium complexity care and served as clinical support for permanent high-complexity hospitals with ICU beds dedicated to COVID-19 patients (17).

São Paulo, Brazil’s largest city, faced an overwhelming surge of COVID-19 cases, with hospitals operating at near-full capacity and significant strain on resources. The rapid increase in cases left the healthcare system vulnerable, prompting the creation of temporary field hospitals. These facilities, like Pacaembu, became crucial in providing care for mild to moderate COVID-19 patients, helping manage the crisis and prevent further escalation of the pandemic. This field hospital allowed for earlier intervention and contributed significantly to reducing pressure on São Paulo’s overwhelmed healthcare system.

Although field hospitals have been implemented in several countries, there remains debate regarding their true cost-effectiveness in responding to health emergencies, and few studies have thoroughly examined this issue (7). For example, in Wuhan, China, 16 Fang Shelter hospitals were constructed within a period of 1 month or more, accommodating approximately 16,000 patients with mild to moderate COVID-19 (18) Cai et al. conducted a retrospective observational study to evaluate the clinical outcomes of 2,011 COVID-19 patients and the associated resource utilization at the Leishenshan Fang Shelter Hospital in Wuhan, China, during its 67 days of operation, from February 8 to April 14, 2020. The overall case fatality rate at Leishenshan during this period was 2.3%, and the average length of stay was 19 days. The estimated total cost of building the facility and treating COVID-19 patients was US$231 million. The average total cost of care was US$114,793, with a direct cost of US$2,288 per patient. The direct cost for critically ill patients was five times higher than for those with mild to moderate disease (US$6,428 vs. US$1,257) (19).

Similarly, an economic analysis of COVID-19 treatment costs was conducted at the largest public general hospital in Latin America, located in São Paulo, Brazil, evaluating both direct and fixed costs. The average cost per admission was US$12,637.42, which increased to US$20,002.80 when considering the need for high-complexity hospital care for COVID-19 patients, with an overall daily cost of US$919.24. These figures can help estimate the cost savings achieved by redirecting COVID-19 patients from general hospitals to field hospitals (20).

In the United Kingdom, the NHS Nightingale Hospital initiative was developed as a field hospital response to the pandemic, with varied objectives: some facilities were set up primarily for critical care or palliative care for patients nearing the end of life, while others were designed to treat mild to moderate COVID-19 cases or provide care for recovering patients. However, few patients were admitted due to several challenges, including logistical difficulties in transporting critically ill patients, a better understanding of the need for high-complexity hospitals for COVID-19 care, the distance of the hospitals from patients, and particularly the shortage of healthcare professionals relative to the number of beds available (21). Similarly, a scoping review highlighted various challenges in managing field hospitals during the COVID-19 pandemic, such as staff shortages, lack of supplies and resources, difficulties in predicting patient numbers, and ineffective communication. Several studies underscored the difficulties in recruiting a large number of qualified professionals with diverse clinical backgrounds and experience for field hospitals (8). In contrast, the Pacaembu Field Hospital did not face issues related to the number of healthcare professionals, as idle professionals from private hospitals were reassigned, and a streamlined approach for hiring and training new staff was implemented.

The experience at the Javits Convention Center in New York provides another perspective. A study involving 1,095 patients treated over 28 days at this temporary facility, which was equipped with 512 general medical beds (organized into 16 pods of 32 beds each) and 48 intensive care unit (ICU) beds with oxygen, monitors, and appropriate staffing, demonstrated the impact of field hospitals. The ability to care for over 100 patients a day during the pandemic peak was crucial in saving many lives. This temporary hospital significantly alleviated pressure on neighboring hospitals by reducing their patient volumes by approximately 10–20% and decreasing emergency department volumes by 10%. These outcomes led to reduced resource utilization, lower staffing needs, and decreased provider stress. Moreover, a well-structured discharge plan with a length of stay of up to 5 days was key to the initiative’s success (22).

In the case of the Pacaembu Field Hospital, the strategy of establishing a temporary facility during the peak of the pandemic to care for non-critical patients, with a focus on ward beds, proved effective in reducing the use of more costly resources. This approach allowed for early intervention for mild to moderate cases at higher risk of clinical deterioration. Additionally, integration within a centralized health system for transfer regulation and bed management facilitated better control and planning across hospital units, contributing to more efficient healthcare delivery.

In Brazil, the threshold for cost-effectiveness is set at a maximum of 0.87 times the Gross Domestic Product (GDP) per capita per year of life gained, which corresponds to R$ 50,193.72 (US$ 9,312.30) in 2023. However, the definition of this threshold is context-specific, depending on local wealth, the characteristics of the healthcare system, the availability of and ability to pay for resources, as well as social preferences. The use of this threshold should always be considered alongside other criteria. Additionally, it is important to note that the costs included in this analysis encompass everything from the construction of the Field Hospital to the full costs of disposing of supplies and personal protective equipment used.

This study has several limitations that should be considered when interpreting the results. Firstly, the analysis covers the early days of the COVID-19 pandemic in 2020, a period before vaccines and specific treatments were available. This lack of therapeutic interventions and the emergency nature of the situation may have significantly impacted clinical outcomes and associated costs, potentially increasing the severity of cases and the cost of medical care. Additionally, socioeconomic factors and regional disparities in healthcare access may have introduced variability in the data that was not fully controlled for. Mortality cases for the entire municipal public network were extrapolated from cases transferred to hospitals managed by SBIBAE, and thus are not based on real-life data. Consequently, the findings may not accurately reflect the actual mortality rates across the entire municipal public network. These limitations should be considered when interpreting the results, as the extrapolated data might not represent the true extent of mortality within the broader network. While the cost-effectiveness analysis of the field hospital offers valuable insights into the financial impact and effectiveness of interventions during the pandemic’s peak, caution is needed when applying these results to future contexts or different epidemiological scenarios.

## Conclusion

The findings from this study underscore the significant role that the Pacaembu Field Hospital played in mitigating the impact of COVID-19 in São Paulo, Brazil. By providing critical care to a large number of patients with mild to moderate symptoms, the hospital effectively reduced mortality rates and contributed substantially to the preservation of life. The cost-effectiveness analysis further highlights the economic value of this intervention, particularly when compared to standard care scenarios in overwhelmed healthcare settings. Despite the inherent challenges and costs associated with establishing field hospitals, the Pacaembu model demonstrated a viable strategy for saving lives during public health emergencies. These results not only validate the utility of field hospitals in crisis situations but also provide a foundation for future planning and resource allocation in similar contexts.

## Data Availability

The raw data supporting the conclusions of this article will be made available by the authors, without undue reservation.

## References

[1] Pan American Health Organization. WHO-PAHO Guidelines for the use of foreign field hospitals in the aftermath of sudden-impact disasters. (2003). Available online at: https://www.who.int/hac/techguidance/pht/FieldHospitalsFolleto.pdf (Accessed on March 24, 2024)

[2] Borrowed Buildings: Canada’s Temporary Hospitals during World War I. Annmarie Adams. Canadian bulletin of. Med Hist. (1999) 16:25–48. doi: 10.3138/cbmh.16.1.25, PMID: 14531398

[3] Hirayama Kensuke Hirota Nobuhiko. Mobile hospital system. U.S. Patent No. 6,179,358. 30. (2001).

[4] Fang D Pan S Li Z Yuan T Jiang B Gan D. Large-scale public venues as medical emergency sites in disasters: lessons from COVID-19 and the use of Fangcang shelter hosp itals in Wuhan, China. BMJ Glob Health. (2020) 5:e002815. doi: 10.1136/bmjgh-2020-002815, PMID: 32546589 PMC7298718

[5] Meira Reis R dos Santos Cirilo Neto S Teixeira Coelho M, Stadiums x Covid-19: a new way to twist. Editora. (2023). Available online at: https://sevenpublicacoes.com.br/index.php/editora/article/view/1981 (Accessed on 2024 Mar. 28)

[6] Chen S Zhang Z Yang J Wang J Zhai X Bärnighausen T. Fangcang shelter hospitals: a novel concept for responding to public health emergencies. Lancet. (2020) 395:1305–14. doi: 10.1016/S0140-6736(20)30744-3, PMID: 32247320 PMC7270591

[7] Li G Du H Fan J He X Wang W. The effect of Fangcang shelter hospitals under resource constraints on the spread of epidemics. Int J Environ Res Public Health. (2023) 20:5802. doi: 10.3390/ijerph20105802, PMID: 37239530 PMC10217850

[8] Hamis AA Md Bukhori AB Heng PP Jane Ling MY Shaharuddin MA Fauzi NAF A. Strategies, challenges and opportunities in the implementation of COVID-19 field hospitals: a scoping review. BMJ Open. (2023) 13:e067227. doi: 10.1136/bmjopen-2022-067227, PMID: 36918252 PMC10015674

[9] SEADE-SP. Sistema Estadual de Análises de dados. Coronavírus. Dados completos. (2023). Available online at: https://www.seade.gov.br/coronavirus/ (Accessed 27 July 2024).

[10] IBGE. Brazilian institute of geography and statistics. (2022) Available online at: https://www.ibge.gov.br/estatisticas/sociais/populacao/9126-tabuas-completas-de-mortalidade.html?edicao=32297&t=resultados (Accessed 27 July 2024).

[11] Lee SW. Methods for testing statistical differences between groups in medical research: statistical standard and guideline of life cycle committee. Life Cycle. (2022) 2:e1. doi: 10.54724/lc.2022.e1

[12] Brazilian Health Ministry. Report on the use of cost-effectiveness thresholds in health decisions. (2022) Available online at: https://www.gov.br/conitec/pt-br/midias/pdf/2022/20221106_relatorio-uso-de-limiares-de-custo-efetividade-nas-decisoes-em-saude.pdf (Accessed 27 July 2024).

[13] Castro Delgado R Pérez Quesada P Pintado García E Marañón Zabalza I Vallina-Victorero Vázquez M Escribano Balín R. Alternate care sites for COVID-19 patients: experience from the H144 hospital of the health service of the principality of Asturias. Spain Prehosp Disaster Med. (2021) 36:774–81. doi: 10.1017/S1049023X21001102, PMID: 34612185 PMC8529347

[14] Yuan Y Qiu T Wang T Zhou J Ma Y Liu X. The application of temporary ark hospitals in controlling COVID-19 spread: the experiences of one temporary ark hospital, Wuhan. China J Med Virol. (2020) 92:2019–26. doi: 10.1002/jmv.25947, PMID: 32343419 PMC7267641

[15] Sacchetto D Raviolo M Beltrando C Tommasoni N. COVID-19 surge capacity solutions: our experience of converting a concert hall into a temporary hospital for mild and moderate COVID-19 patients. Disaster Med Public Health Prep. (2022) 16:1273–6. doi: 10.1017/dmp.2020.412, PMID: 33100254 PMC7943956

[16] Spagnolello O Rota S Francesco Valoti O Cozzini C Parrino P Portella G. Bergamo field hospital confronting COVID-19: operating instructions. Disaster Med Public Health Prep. (2022) 16:875–7. doi: 10.1017/dmp.2020.447, PMID: 33208198 PMC7985639

[17] da Saúde Ministério. Cadastro Nacional de Estabelecimentos de Saúde (CNES). Brasília (2024). Available online at: https://cnes.datasus.gov.br/ (Accessed 20 June 2024).

[18] Liu P Zhang H Long X Wang W Zhan D Meng X. Management of COVID-19 patients in Fangcang shelter hospital: clinical practice and effectiveness analysis. Clin Respir J. (2021) 15:280–6. doi: 10.1111/crj.13293, PMID: 33051994 PMC7675548

[19] Cai Y Chen Y Xiao L Khor S Liu T Han Y. The health and economic impact of constructing temporary field hospitals to meet the COVID-19 pandemic surge: Wuhan Leishenshan Hospital in China as a case study. J Glob Health. (2021) 11:05023. doi: 10.7189/jogh.11.05023, PMID: 34912549 PMC8645218

[20] Miethke-Morais A Cassenote A Piva H Tokunaga E Cobello V Rodrigues Gonçalves FA. COVID-19-related hospital cost-outcome analysis: the impact of clinical and demographic factors. Braz J Infect Dis. (2021) 25:101609. doi: 10.1016/j.bjid.2021.101609, PMID: 34454894 PMC8373618

[21] KINGSFUND. NHS nightingale hospitals worth money. (2021) Available online at: https://www.kingsfund.org.uk/blog/2021/04/nhs-nightingale-hospitals-worth-money (Accessed October 4th, 2022)

[22] Brady K Milzman D Walton E Sommer D Neustadtl A Napoli A. Uniformed services and the field hospital experience during Coronovirus disease 2019 (SARS-CoV-2) pandemic: open to closure in 30 days with 1,100 patients: the Javits New York Medical Station. Mil Med. (2022) 187:e558–61. doi: 10.1093/milmed/usab003, PMID: 33580799 PMC7928760

